# Research on the Evaluation of Cross-Border E-Commerce Overseas Strategic Climate Based on Decision Tree and Adaptive Boosting Classification Models

**DOI:** 10.3389/fpsyg.2021.803989

**Published:** 2021-12-23

**Authors:** Yi Lei, Xiaodong Qiu

**Affiliations:** School of Economics and Management, Beijing Jiaotong University, Beijing, China

**Keywords:** cross-border e-commerce, strategic climate, “Belt and Road” countries, machine learning, Decision Tree, Adaptive Boosting

## Abstract

At present, China’s cross-border e-commerce has ushered in a golden period of development. When developing cross-border e-commerce, enterprises should first assess the market climate of the target country and reasonably select the target country. Based on the PESTEL theory, an evaluation index system is established for China’s cross-border e-commerce overseas strategic climate. Taking “One Belt, One Road” as the opportunity and background, the overseas strategic climate of cross-border e-commerce in 62 countries along the “One Belt, One Road” is selected as the research object, and the Decision Tree and Adaptive Boosting classification methods in machine learning are applied to train and predict the established index system. Finally an overall picture of the overseas strategic climate of the 62 countries is obtained. The results are compared and analysed in depth to identify the most suitable countries for cross-border e-merchants and to provide reference for cross-border e-merchants investors.

## Introduction

E-commerce has developed rapidly as an efficient business model (Wang et al., 2021). Especially this year, under the impact of the global epidemic, e-commerce has played an important role in counteracting the impact of the epidemic, promoting consumer recovery and driving global economic recovery with its ability to break through time and space constraints, reduce intermediate links and resolve information asymmetry between supply and demand. As the country with the largest scale of e-commerce, China has a mature logistics system, an aggregated manufacturing industry chain, an efficient operation model, a specialised platform and a relatively well-established business ecosystem, and has a first-mover advantage in areas such as logistics, manpower, and payment (Lei and Qiu, 2020). As Chinese e-commerce has become relatively mature in the domestic market, it has turned to apply its rich experience and mature development system to the international market, and cross-border e-commerce has thus grown rapidly. The State also attaches great importance to the development of cross-border e-commerce in China. Since 2013, the Central Committee of the Communist Party of China, the State Council and relevant departments have issued a number of documents to enhance and speed up the development of cross-border e-commerce in China in terms of cross-border trade RMB settlement, trade taxation, customs supervision and inspection, simplification of systems and processes, and construction of free trade zones.

In 1994, Amazon was established, and in 2012 officially launched “global selling.” Under the influence of this, China’s cross-border e-commerce began an important transformation path. Although China’s history of cross-border e-commerce development is short com-pared to that of developed countries, it is developing at an extremely fast pace and the scale of development is increasing with the efforts of all parties. In 2020, China’s cross-border e-commerce imports and exports reach 1.69 trillion yuan, with a growth rate of 31.1%, of which exports are 1.12 trillion yuan, an increase of 40.1%.^1^ It is evident that the global demand for Chinese products is expanding and the market is highly competitive, therefore the comprehensive development of cross-border e-commerce and the establishment of a complete ecosystem will be an inevitable trend in the future and China’s cross-border e-commerce will usher in a new golden age of development.

The external macro environment is a prerequisite for the existence of a business and has a direct role in driving, constraining, and interfering with the continued development of the business (Raphaël et al., 2004). To develop cross-border e-commerce, we must first make a good judgement of the external environment, reasonably select the target market and conduct relevant strategic analysis in order to make cross-border e-commerce more efficient out of the country and take root in the international market.

Based on the above background, this study establishes an evaluation index system for the external strategic climate of cross-border e-commerce in China based on relevant theories, and makes a reasonable assessment of the offshore marketing environment. The established indicator system is predicted and evaluated by machine learning methods which are Decision Tree model and Adaptive Boosting model (AdaBoost). The learning results are compared and analysed to guarantee the accuracy of the results and to provide a basis for finding economies with an advantageous external climate for cross-border e-commerce.

## Literature Review

### Research on Cross-Border E-Commerce

Since 2013, research on cross-border e-commerce has attracted widespread attention, and after 2016, there has been an explosive growth in related research. With the upgrading of consumption and the increasing pursuit of a better life by domestic consumers, cross-border e-commerce has ushered in an era of great development (Zhang et al., 2021). Cross-border e-commerce is of strategic importance as a technological basis for promoting economic integration and trade globalisation (Jiang and Ma, 2021). Cross-border e-commerce not only breaks down the barriers between countries and makes international trade borderless, but also brings great changes to the world economy and trade (Li, 2020). Among all research scholars, Chinese scholars occupy the vast majority, while others mainly come from the United States, Germany, South Korea, etc.

In terms of research content, it is mainly divided into macro studies and related technical-level studies. The macroscopic studies are mainly qualitative studies, which focus on the development of cross-border e-commerce. They argue that the development of cross-border e-commerce cannot be separated from a perfect legal system and infrastructure construction (Kawa and Zdrenka, 2016; Aqlan, 2020). Most of the studies on the technical aspects of cross-border e-commerce are mainly quantitative, including mainly the studies related to logistics system and talent training. Kumar et al. (2018) argued that the employability of talents cultivated by cross-border e-commerce industry-university research is related to pre-vocational training. Wu and Clin (2018) analysed the problems in cross-border e-commerce talent training, and then proposed countermeasures for cross-border e-commerce talent training in vocational colleges under the integration of industry-education model. Some scholars studied the role of logistics for cross-border e-commerce and considered logistics as critical (Giuffrida et al., 2017; Li, 2018). Asch et al., 2020) studied the cross-border e-commerce market from the perspective of airports and considered pre-clearance capacity, air and land transportation capacity as prerequisites for the development of cross-border e-commerce.

### Indicator System Evaluation Methodology

When choosing a method for the evaluation of the indicator system, different methods can be chosen according to the evaluation object and the purpose of the evaluation. For the determination of indicator weights, there are mainly subjective assignment methods and objective assignment methods. The subjective empowerment method includes the Delphi method and the hierarchical analysis method. The Delphi method is a subjective evaluation and forecasting method that repeats the process of expert consultation, with the aim of integrating the opinions of all participating experts and eventually reaching a consensus (García-Aracil and Palomares-Montero, 2012). Hierarchical analysis (AHP) is used for systems with a multi-level structure, and the comparison of relative quantities is used to take the feature vector corresponding to its feature roots as weights. It uses a two-by-two comparison of attributes to calculate weights, integrates the opinions of several experts, eliminates some obviously unreasonable data, and has a certain degree of objectivity (Beskese and Bozbura, 2014). The main objective weighting methods are the entropy method (Sun et al., 2017), the coefficient of variation method (Qi et al., 2021) and the correlation coefficient method (Abdelmoaty et al., 2021). When more complete sample data is available, objective weighting can be used and the results can be modified by cross-sectional comparisons between indicators. In the absence of sample data, especially when there are a large number of qualitative indicators, the subjective weighting method can be used. For the comprehensive evaluation of indicator system models, the main methods are principal component analysis (Spencer et al., 2020; Fayed and Jiazhu, 2021), factor analysis (Luo et al., 2020), cluster analysis (Hallak et al., 2013; Zhong et al., 2018), grey correlation (Chen et al., 2009), and so on.

### Machine Learning Applications

Machine learning has been integrated into agriculture, industry, service industry, and many derivative industries over a certain period of time. In recent years, in the era of rapid Internet development and the emergence of massive amounts of data, scholars have started to study some new models to adapt to the big data context (Hoffman et al., 2010; Agarwal et al., 2014). At present, it can almost be considered that big data is the best scenario for machine learning applications, and machine learning has become an important branch of artificial intelligence. Today, machine learning has gradually become an indispensable solution in the industry, profoundly changing human life. The most mature application direction is computer vision, i.e., imitating the human eye to identify, track and measure targets, etc. Applications in image processing in the medical field and product quality control in industry are particularly prominent (Vanneste et al., 2021). Data visualisation of human body systems using computer vision techniques can help to personalise patient care (Razeghi et al., 2020). Computer vision is often used in conjunction with speech recognition and has been well used in speech translation speech recognition in the Internet domain (Gray and Suzor, 2020). Speech recognition has been expanded to form a new field of machine learning, namely natural language processing, which is mainly used in text processing such as machine translation, opinion monitoring, and text classification (He, 2018). Pattern recognition is a classic application area of machine learning, a process of perceiving, processing, and making judgements, often used in traffic surveying (Zhen et al., 2020). Statistical learning, big data analysis and data mining are machine learning fields that need to be used in combination with big data, which can be used to explore the potential value behind the data through readily available data, and are often used for promotion in the Internet field and analysis of the securities market in the financial field (Khan et al., 2020).

For the application of machine learning in prediction, regression algorithms are usually used for prediction. Most scholars base their research on the user perspective. Analysis of users’ behaviour and the herding among them can better predict their future behaviour (David et al., 2003); consumer preferences can be predicted based on the content of keywords and purchase behaviour entered by consumers (Kim et al., 2001; Liu and Toubia, 2018); and companies can predict user churn by tracking consumers’ behaviour related to emails (Ascarza et al., 2018). In addition, the prediction of app ad conversions (Olivier et al., 2014) and e-commerce transactions (Sheikh and Ebrahim, 2017) have also received attention from scholars.

Through a literature review of the research on cross-border e-commerce, the evaluation methods of strategic climate evaluation index systems and machine learning application areas, it is found that the current research direction is mainly based on cross-border e-commerce itself, which mainly focuses on cross-border e-commerce industry chain links and related peripheral needs. However, cross-border e-commerce is global and transnational, and different countries are bound to have different external environments, such as legal, political, and cultural, which often play an important role in the development of cross-border e-commerce. And the existing research methods are generally the existing traditional methods, and because the evaluation system examines a wide range of factors and lacks relatively unified basic indicators and basic algorithms, the results obtained by different evaluation methods are greatly different. Moreover, scholars usually apply only one evaluation method to the evaluation of an indicator system, focusing only on the evaluation results but neglecting the rationality of the evaluation process, resulting in questionable evaluation results. Moreover, there is almost no research on the application of machine learning to strategic climate evaluation. This study combines machine learning with the assessment of the cross-border e-commerce offshore market climate, breaking away from the traditional approach to the evaluation of indicator systems and opening up new areas of research in cross-border e-commerce and application of machine learning. Two machine learning methods are used in this study to avoid random errors and guarantee the accuracy of the results.

## Materials and Methods

### Materials

The PESTEL theory, also known as the grand environmental analysis theory, adds environmental and legal factors to the PEST. Many scholars have conducted strategic research based on this theory (Wei et al., 2019; Kamile et al., 2020). Based on the PESTEL theory, this study establishes an evaluation index system for the external strategic climate of cross-border e-commerce from six aspects: political factors, economic factors, social factors, techno-logical factors, environmental factors, and legal factors.

Politics is a prerequisite for determining open cooperation between countries. A stable political environment plays a vital role in safeguarding the development of cross-border e-commerce, especially in volatile countries, which are sometimes disturbed by the political environment. Changes in the economic environment have a direct impact on cross-border e-commerce, and its condition directly affects the earnings of enterprises; the economy is a prerequisite for conducting trade. Social factors mainly refer to the local people’s awareness of cross-border e-commerce, consumption patterns, and the cultural quality of the population. The social environment affects the scale of cross-border e-commerce development. The technological environment indirectly or directly affects the efficiency of cross-border e-commerce. Ease of transportation, logistics timeliness and internet penetration have the most direct impact on the consumer experience. Environmental factors affect the speed of cross-border e-commerce development. Good industry development trends and sustainable development space can greatly facilitate the development of cross-border e-commerce. A sound legal environment can keep every link in the industry chain functioning well by regulating, restricting and maintaining the links.

Based on the above construction ideas, according to the principles of scientific validity, accessibility, applicability, etc., of data, this study has constructed a cross-border e-commerce overseas strategic climate evaluation index system containing three dimensions, as shown in Table 1. Scientific and applicability means that the selected indicators should be suitable for cross-border e-commerce, can effectively represent the external strategic climate, and the data sources are accurate, real and valid. Among the indicators of the same category, those with strong representativeness are selected to reflect other similar indicators. Accessibility means that there should be a way to source data. The lack of data on some Belt and Road countries has a great influence on the selection of evaluation indicators. In order to ensure the reliability of the evaluation indicators, this study mainly selects the relevant indicators from the world authoritative database. The selection of indicators should also be systematic and hierarchical to ensure that the overall situation can be comprehensively evaluated and the depth of evaluation can be reflected. Based on this, the evaluation index system of cross-border e-commerce overseas strategic climate is constructed. The primary indicators are political factors, economic factors, social factors, technological factors, environmental factors, and legal factors, including 13 secondary indicators and 26 tertiary indicators.

**TABLE 1:** Indicator system for evaluating the overseas strategic climate of cross-border e-commerce.

Primary indicator	Secondary indicator	Three-level indicator
Political factors	Government execution	Government accountability X_1_
		Government efficiency X_2_
		Supervision quality X_3_
	Political stability	Political stability, absence of violence X_4_
Economic factors	Economic strength	Total GDP X_5_
		GDP growth rate X_6_
		Per capita GDP X_7_
	Economic stability	Inflation (measured by consumer price index) X_8_
	Economic openness	Foreign direct investment X_9_
		Dependence on foreign trade X_10_
Social factors	Demographic environment	Total population X_11_
		Proportion of the population living in poverty X_12_
		Number of people aged 15–64 X_13_
	Cultural background	Salaried women as a proportion of female employment X_14_
		Percentage of unemployed X_15_
Technological factors	Telecommunication condition	Mobile cellular subscriptions per 100 people X_16_
		Number of secure internet servers X_17_
		Internet penetration rate X_18_
	Logistics conditions	Railway (total kilometres) X_19_
		Air outbound traffic X_20_
		Container terminal throughput X_21_
Environmental factors	Industry development	Imports of goods and services as a percentage of GDP X_22_
		Annual growth rate of imports of goods and services X_23_
	Sustainability	PM2.5 air pollution rate annual average exposure X_24_
Legal factors	Citizenship	Law-ruled environment X_25_
	Laws and regulations	Legal power index X_26_

The indicator data in the overseas strategic climate evaluation index system are obtained from the world authoritative database, among which X_1–_X_4_ and X_25–_X_26_ indicator data are obtained from the World Governance Indicators (WGI) report released by the World Bank, and other indicators or the original calculation data of indicators are obtained from the World Bank database. For the missing data, the commonly used average method is used for processing.

**FIGURE 1:**
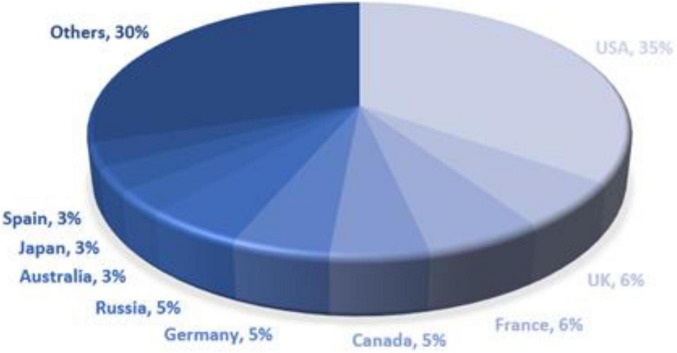
China export parcels August 2019–July 2020.

The data includes “with label” and “without label” data. The data with labels is “natural” data, and the top nine countries in terms of the percentage of China’s export destinations from August 2019 to July 2020 are selected as positive data with labels, namely Australia, Canada, Germany, Spain, France, the United Kingdom, Japan, Russia, and the United States, see Figure 1 for details. The strategic climate for e-commerce is good and conducive to cross-border e-commerce presence. African countries are rapidly developing their communications infrastructure, with the highest number of internet users in Nigeria, followed by Egypt, Kenya, South Africa, and Morocco. African e-commerce giant Jumia’s sites are also mostly established in these countries, in addition to Côte d’Ivoire. However, cross-border e-commerce export packages in these African countries are low, so the countries of Cote d’Ivoire, Nigeria, Kenya, South Africa, and Morocco are chosen to be included in the negative band label data. The indicator data of these countries for the last 10 years are selected for training and prediction.

Since the introduction of China’s “Vision and Action for Promoting the Construction of the Silk Road Economic Belt and the 21st Century Maritime Silk Road” (the “Belt and Road”) in March 2015, China is gradually strengthening global openness and cooperation, actively promoting the building of interconnected partnerships with various countries along the route; it is strengthening innovative trade methods and we are building a win–win economic cooperation framework for all countries along the route to achieve common development and prosperity. The “Belt and Road” initiative is a new form of international economic cooperation between China and its new partners (Huang, 2016), a model for sustainable development (Michael and Wei, 2019), which can achieve international economic, political, and cultural development through diversified communication and exchange under the principle of peaceful coexistence between countries, and is important for promoting economic growth. It is an important contribution to the restructuring of the international economic system. Currently, some scholars have studied the impact of the Belt and Road Initiative on a particular economy, such as the Middle East (Kamel, 2018), Europe (Garcia and Xu, 2017), Africa and Central Asia (Huang et al., 2019), arguing that the Belt and Road Initiative will create a mutually beneficial and the “One Belt, One Road” initiative will create a mutually beneficial and interconnected cooperation platform that will bring huge economic development opportunities for China and countries along the route. In addition to the promotion of good policies of the Belt and Road, the opening of the China-Europe Class Train has become an important hub of the “One Generation, One Road” (Dmitri et al., 2020), providing great convenience for cross-border e-commerce to reduce costs and open up markets in various countries. Therefore, this study takes “One Belt, One Road” as the opportunity and background, relying on good national policies, and selects the strategic market climate of countries along the “One Belt, One Road” for forecasting, which is of practical guidance for the active development of cross-border e-commerce in China. Depending on availability, these unlabelled data include 62 countries which are Afghanistan, Albania, United Arab Emirates, Armenia, Azerbaijan, Bangladesh, Bulgaria, Bahrain, Bosnia and Herzegovina, Belarus, Brunei, Bhutan, Czechia, Egypt, Estonia, Georgia, Croatia, Hungary, Indonesia, India, Iran, Iraq, Israel, Jordan, Kazakhstan, Kyrgyzstan, Cambodia, Kuwait, Laos, Lebanon, Sri Lanka, Lithuania, Latvia, Moldova, Maldives, North Macedonia, Myanmar, Montenegro, Mongolia, Malaysia, Nepal, Oman, Pakistan, Philippines, Poland, Qatar, Romania, Saudi Arabia, Singapore, Serbia, Slovakia, Slovenia, Syria, Thailand, Tajikistan, Turkmenistan, Timor-Leste, Turkey, Ukraine, Uzbekistan, Vietnam, and Yemen, of which 42 are in Asia, 19 in Europe and 1 in Africa. Data for the last 10 years for these countries are selected for projections.

### Methods

Machine learning methods can be used purely from indicator data to conduct holistic, open-ended and global analysis and evaluation to find out the inner logic between indicator data and effectively predict the indicator system, which plays an important role in building an evaluation indicator system with wider applicability and higher accuracy. At the same time, it can break the traditional cognition and guide further development strategies and choices by identifying the intrinsic link between indicator data and prediction results. According to the requirements, the machine learning models chosen for this article are Decision Tree model and AdaBoost. These two models are chosen because compared to other models, the Decision Tree model is easier to prepare data, can produce very good results for a large amount of data in a shorter period of time, and the Decision Tree is easy to understand. The AdaBoost model is highly accurate, simple to construct, and easy to understand and code. Both methods can achieve the purpose of the study, while the results can be compared. In this study, the language tool applied is python.

The Decision Tree is a model for classifying instances based on input features, which are conditional probability distributions defined over a feature space and a class space. The Decision Tree method has been widely used as a decision making technique in corporate investment decisions, most notably in the study of the spread of panic in interpersonal networks (Kelly and Grada, 2000) and in making causal inferences (Athey and Imbens, 2015). Decision Tree is one of the most common and popular methods of stochastic decision modelling, which can assess the importance of features, predict the true state of the evaluation target and effectively control risk (Panigrahi et al., 2021).

**FIGURE 2:**
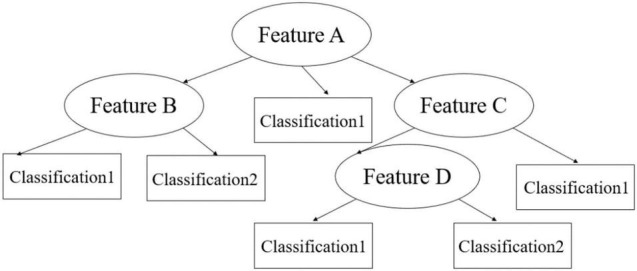
Principle of Decision Trees.

The principle of the Decision Tree is similar to that of a tree structure, consisting of decision nodes, branches, and leaves as shown in Figure 2 below. Each internal node represents a judgement on an attribute, each branch represents the output of a judgement, and finally each leaf node represents a classification result. The advantages of the Decision Tree are that the results are visual, easy to understand and relatively low computational effort.

**FIGURE 3:**
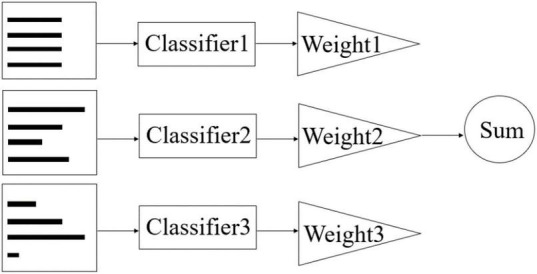
Principle of AdaBoost.

The core idea of AdaBoost is to generate different weak classifiers for the same training set, and then collect these classifiers to form a stronger classifier to achieve a boosting process of the original classifier, which is shown in Figure 3 below. The key of this algorithm is to adjust the weights of each sample in the training set, which has the advantage of significantly improving the prediction accuracy of sub-classifiers without requiring a priori knowledge and solid theory, and therefore has received attention from researchers in different fields and has been widely used in various industries, such as recognition (Yan and Luo, 2012), localisation (Cheng et al., 2008), and classification (Nizamani et al., 2011).

This study uses a classification and regression tree (CART), which is a learning method that outputs a conditional probability distribution of a random variable *Y* given an input random variable *X*. There are only two choices of nodes. The feature space is divided into a finite number of cells by continuous partitioning, and the predicted probability distribution is determined over these cells. CART uses the Gini index to select the optimal cut-off feature, and because the basic structure of a binary tree is used, each partition is bifurcated. Gini index defined:

(1) $$G\,i\,n\,i\,\left(X\right)=\sum_{i=1}^{k}p_{i}\,\left(1-p_{i}\right)=1-\sum_{i=1}^{k}p_{i}^{2}$$

*K*, number of indicators; *X*, random variable; *p_*i*_*-random variables corresponding to probabilities *p_1_*, *p_2_*,…, *p_*pk*_*

If the sample set D is partitioned into two parts D_1_ and D_2_ according to whether feature A takes a certain value a:

(2) $$D_{1}=\left(x,y\right)\in D|A\,\left(x\right)=a_{,}\,D_{2}=D-D_{1}$$

Then, conditional on characteristic A, the Gini index of the set D is defined as:

(3) $$i\,n\,i\,\left(D,A\right)=\frac{\left|D_{1}\right|}{\left|D\right|}\,G\,i\,n\,i\,\left(D_{1}\right)+\frac{\left|D_{2}\right|}{\left|D\right|}\,G\,i\,n\,i\,\left(D_{2}\right)$$

The Gini index Gini(D) represents the uncertainty of D. The Gini index Gini(D, A) represents the uncertainty of the set D after the partition of A = a. The larger the value of the Gini index, the greater the uncertainty of the sample set, similar to entropy.

## Results

A total of 155 samples of “tagged” data are first trained and tested in a ratio of 7:3, with “tagged” representing a good or bad strategic environment for cross-border e-commerce in the country, as “Yes (Y)” or “No (N).” This is done to validate the accuracy of the model. A part of the data is taken out for training and another part is used for testing. When the model is trained, it must match the distribution of the training data. In order to verify the generalisation ability of the model, the data set that is not involved in the training is put into the model for testing, and the accuracy of the model prediction can be ensured by comparing it with the real value. After that, the valid model can be used to predict the data to ensure the validity of the results. The prediction accuracy of the two models is over 95% in the past 10 years, which fully demonstrates the effectiveness of the model.

**FIGURE 4:**
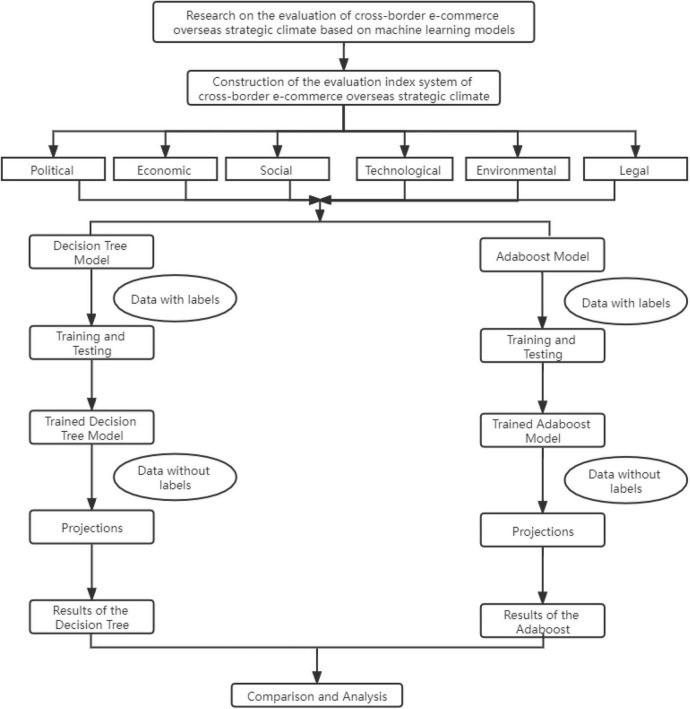
Diagram of the analysis process.

The trained model is then used to predict the “un-labelled” data to assess the strategic climate for cross-border e-commerce in Belt and Road countries. A total of 693 samples are predicted and the results are finally tallied. This method ensures that the results are objective and accurate. The prediction results of the two models are compared and found to be identical, which fully guarantees the accuracy of the prediction results. In order to represent the analysis process more clearly, a diagram is made as in Figure 4.

The final results are divided into three categories. The first category is for countries with “N” prediction results in the past 10 years, with 25 countries in total, where the strategic climate for cross-border e-commerce has been unsatisfactory in the past 10 years, as shown in Table 2. The second group of countries with “Y” for the last 10 years, 26 in total, have a good strategic climate for cross-border e-commerce in the last 10 years, as shown in Table 3. There are also 11 countries with both “N” and “Y” for the last 10 years, and these countries have had an unstable and fluctuating strategic climate for cross-border e-commerce over the last 10 years, as shown in Table 4.

**TABLE 2:** Countries with “N” forecast for the last 10 years.

No.	Country	Year									
		10	11	12	13	14	15	16	17	18	19
1	Afghanistan	N	N	N	N	N	N	N	N	N	N
2	Albania	N	N	N	N	N	N	N	N	N	N
3	Armenia	N	N	N	N	N	N	N	N	N	N
4	Egypt	N	N	N	N	N	N	N	N	N	N
5	Bangladesh	N	N	N	N	N	N	N	N	N	N
6	Kyrgyzstan	N	N	N	N	N	N	N	N	N	N
7	Cambodia	N	N	N	N	N	N	N	N	N	N
8	Georgia	N	N	N	N	N	N	N	N	N	N
9	Indonesia	N	N	N	N	N	N	N	N	N	N
10	India	N	N	N	N	N	N	N	N	N	N
11	Jordan	N	N	N	N	N	N	N	N	N	N
12	Sri Lanka	N	N	N	N	N	N	N	N	N	N
13	Moldova	N	N	N	N	N	N	N	N	N	N
14	North Macedonia	N	N	N	N	N	N	N	N	Y	Y
15	Burma	N	N	N	N	N	N	N	N	N	N
16	Mongolia	N	N	N	N	N	N	N	N	N	N
17	Nepal	N	N	N	N	N	N	N	N	N	N
18	Pakistan	N	N	N	N	N	N	N	N	N	N
19	Philippines	N	N	N	N	N	N	N	N	N	N
20	Tajikistan	N	N	N	N	N	N	N	N	N	N
21	East Timor	N	N	N	N	N	N	N	N	N	N
22	Ukraine	N	N	N	N	N	N	N	N	N	N
23	Uzbekistan	N	N	N	N	N	N	N	N	N	N
24	Vietnam	N	N	N	N	N	N	N	N	N	N
25	Yemen	N	N	N	N	N	N	N	N	N	N

*Y/N indicates good/bad overseas strategic climate for cross-border e-commerce.*

**TABLE 3:** Countries with “Y” forecast for the last 10 years.

No.	Country	Year									
		10	11	12	10	14	15	10	17	18	10
1	Czechia	Y	Y	Y	Y	Y	Y	Y	Y	Y	Y
2	Estonia	Y	Y	Y	Y	Y	Y	Y	Y	Y	Y
3	Croatia	Y	Y	Y	Y	Y	Y	Y	Y	Y	Y
4	Hungary	Y	Y	Y	Y	Y	Y	Y	Y	Y	Y
5	Israel	Y	Y	Y	Y	Y	Y	Y	Y	Y	Y
6	Kazakhstan	Y	Y	Y	Y	Y	Y	Y	Y	Y	Y
7	Bulgaria	Y	Y	Y	Y	Y	Y	Y	Y	Y	Y
8	Bahrain	Y	Y	Y	Y	Y	Y	Y	Y	Y	Y
9	Kuwait	Y	Y	Y	Y	Y	Y	Y	Y	Y	Y
10	United Arab Emirates	Y	Y	Y	Y	Y	Y	Y	Y	Y	Y
11	Lebanon	Y	Y	Y	Y	Y	Y	Y	Y	Y	Y
12	Lithuania	Y	Y	Y	Y	Y	Y	Y	Y	Y	Y
13	Latvia	Y	Y	Y	Y	Y	Y	Y	Y	Y	Y
14	Maldives	Y	Y	Y	Y	Y	Y	Y	Y	Y	Y
15	Montenegro	Y	Y	Y	Y	Y	Y	Y	Y	Y	Y
16	Malaysia	Y	Y	Y	Y	Y	Y	Y	Y	Y	Y
17	Oman	Y	Y	Y	Y	Y	Y	Y	Y	Y	Y
18	Poland	Y	Y	Y	Y	Y	Y	Y	Y	Y	Y
19	Qatar	Y	Y	Y	Y	Y	Y	Y	Y	Y	Y
20	Romania	Y	Y	Y	Y	Y	Y	Y	Y	Y	Y
21	Saudi Arabia	Y	Y	Y	Y	Y	Y	Y	Y	Y	Y
22	Singapore	Y	Y	Y	Y	Y	Y	Y	Y	Y	Y
23	Slovakia	Y	Y	Y	Y	Y	Y	Y	Y	Y	Y
24	Slovenia	Y	Y	Y	Y	Y	Y	Y	Y	Y	Y
25	Syria	Y	Y	Y	Y	Y	Y	Y	Y	Y	Y
26	Turkey	Y	Y	Y	Y	Y	Y	Y	Y	Y	Y

*Y/N indicates good/bad overseas strategic climate for cross-border e-commerce.*

**TABLE 4:** Countries with both “N” and “Y” forecast for the last 10 years.

No.	Country	Year									
		10	11	12	10	14	15	10	17	18	10
1	Azerbaijan	N	Y	Y	Y	Y	N	N	N	N	N
2	Bosnia and Herzegovina	N	N	N	N	N	N	N	N	Y	Y
3	Belarus	Y	Y	Y	Y	Y	Y	N	N	Y	Y
4	Brunei	Y	Y	Y	Y	Y	Y	N	N	Y	Y
5	Bhutan	N	N	N	N	N	N	N	N	Y	Y
6	Iran	Y	Y	Y	Y	N	N	N	N	Y	Y
7	Iraq	N	Y	Y	Y	Y	N	N	N	N	Y
8	Laos	N	N	N	N	N	N	N	N	Y	Y
9	Thailand	N	N	N	Y	Y	N	Y	Y	Y	Y
10	Turkmenistan	N	N	Y	Y	Y	Y	Y	Y	Y	Y
11	Serbia	N	Y	Y	Y	Y	N	N	Y	Y	Y

*Y/N indicates good/bad overseas strategic climate for cross-border e-commerce.*

## Discussion

According to the above results, 12 European countries, accounting for 63% of the European countries selected for this study, have “Y” prediction in the last 10 years: Czechia, Estonia, Croatia, Hungary, Bulgaria, Lithuania, Latvia, Montenegro, Poland, Romania, Slovakia, and Slovenia. All of these countries signed the China–CEE Cooperation Mechanism (16 + 1 cooperation) with China in 2012. In recent years, China has worked with them to build a new type of cooperation platform, resulting in a number of sizeable, influential, and effective projects. The mechanism has become an important platform for open, inclusive, mutually beneficial and win–win cross-regional cooperation, and its international influence has continued to emerge, promoting the development of bilateral relations between China and CEE countries as well as China–Europe relations. The level of economic and trade cooperation between China and the CEE countries is also rising, with investment expanding and in more diverse forms, involving handicrafts, machinery, finance, and many other fields. At the same time, with the opening of the China–Europe train, the pace of cooperation and construction in the field of infrastructure interconnection has accelerated, some key transportation projects have been steadily promoted, and the role of Central and Eastern European countries as hubs in the Asia–Europe Continental Bridge has been continuously enhanced. Therefore, for these 12 European countries, their cross-border e-commerce offshore strategic climate is relatively forward, their economies are more open, their industry environment is good and their market potential is large, creating a favourable environment for cross-border e-commerce investors. China’s cross-border e-commerce business can be a key consideration when investing. There are 14 other Asian countries with a “Y” forecast in the last 10 years, namely Israel, Kazakhstan, Bahrain, Kuwait, United Arab Emirates, Lebanon, Maldives, Malaysia, Oman, Qatar, Saudi Arabia, Singapore, Syria, and Turkey. Although the strategic climate of these countries is suitable for cross-border e-commerce, it is varies greatly in terms of characteristics, and there is a greater need to enhance the inclusiveness of connectivity.

Twenty of the countries with “N” in the last 10 years are in Asia, accounting for 47% of the Asian countries selected for this study: Afghanistan, Armenia, Bangladesh, Kyrgyzstan, Cambodia, Georgia, Indonesia, India, Jordan, Sri Lanka, Myanmar, Mongolia, Nepal, Pakistan, Philippines, Tajikistan, Timor-Leste, Uzbekistan, Vietnam, and Yemen. There is also one African country, Egypt, and four European countries, Albania, Moldova, North Macedonia, and Ukraine. Most of these countries are developing countries with relatively low levels of economic development. Most countries lack good and accessible infrastructure and existing government planning is inadequate, so the openness and industry environment is relatively weak. Some of the countries also have political disputes and territorial disputes, making the political environment unstable and adding risk to e-commerce investments, so cross-border e-commerce businesses need to con-sider these countries carefully when selecting them.

Three of the countries with both “N” and “Y” in the 10-year forecast are in Europe, namely Bosnia and Herzegovina, Belarus, and Serbia. The remaining eight countries are Asian countries, including Azerbaijan, Brunei, Bhutan, Iran, Iraq, Laos, Thailand, and Turkmenistan. The overall offshore strategic climate for cross-border e-commerce in these countries is unstable, with weak ability to be disturbed by various indicators, and requires a high level of judgement and insight from investors. Therefore, these countries are suitable for those experienced cross-border e-commerce investors to choose from.

## Conclusion

This article establishes an evaluating index system for the overseas strategic climate of cross-border e-commerce. Using the Decision Tree model and the AdaBoost model in machine learning, we analyse the overall situation of the cross-border e-commerce overseas strategic climate of 62 countries along the Belt and Road in the past 10 years based on the scientific, comprehensible and accessible data, combining theoretical and empirical dimensions. The following key findings are obtained.

First, the Decision Tree model and the AdaBoost model to the evaluation of the cross-border e-commerce overseas strategic climate index system are applied. The prediction accuracy rate is above 95% in the last 10 years of data and the prediction results of both models are exactly the same, which shows that machine learning is applicable to the index system established in this study, and the accuracy and validity are guaranteed.

Secondly, the prediction results through machine learning methods are divided into three categories. The first category contains 25 countries with a less than optimal strategic climate for cross-border e-commerce in the last 10 years, all with a prediction result of “N.” The second category contains 26 countries with a good strategic climate for cross-border e-commerce in the last 10 years, all with “Y” prediction. There are also 11 countries with both “N” and “Y” in the 10-year period, where the overseas strategic climate for cross-border e-commerce has been unstable over the last 10 years, with good and bad results. Cross-border e-commerce investors can invest based on the results selectively.

Thirdly, among the countries along the Belt and Road, most European countries have a good and stable overseas strategic climate for cross-border e-commerce. The Czechia, Estonia, Croatia, Hungary, Bulgaria, Lithuania, Latvia, Montenegro, Poland, Romania, Slovakia, and Slovenia are representative of these countries, which have good infrastructure, open economies and a good industry environment, bringing opportunities for cross-border e-commerce investors.

Most of the existing methods for evaluating the offshore strategic climate are subjective analysis or traditional mathematical methods. By establishing an indicator system and combining machine learning with the evaluation of the offshore strategic climate of cross-border e-commerce, this article breaks through the traditional method of evaluating indicator systems and opens up a new method of evaluating the offshore strategic climate of cross-border e-commerce and a new field of application of machine learning. This makes the evaluation process more rational and scientific. This is the innovation of this article and a new attempt. Of course, there are other models of machine learning than the Decision Tree model and the AdaBoost model. The Decision Tree model has the advantages of requiring little background knowledge, low computational complexity and strong explanation, the AdaBoost model makes good use of weak classifiers for cascading and has advantages such as high accuracy, so they are chosen for this study.

In future research, other models of machine learning can be tried and the results obtained from different models can be compared. Moreover, there are many types of goods sold in e-commerce, and different industries may require different external investment climate. The investment strategies needed to target consumer groups with different characteristics may also differ. Researchers may consider further research by industry, merchandise category, and target consumer characteristics.

## Data Availability Statement

The original contributions presented in the study are included in the article/supplementary material, further inquiries can be directed to the corresponding author.

## Ethics Statement

The authors of this article, YL and XQ, clarify that our protocol does not require ethical approval or consent according to our local legislation and national guidelines. This study did not need to conduct the Bioethics (animal subjects), and all the respondents are voluntarily and did not need the clinical trials. Therefore, we believe that an ethical review process was not required for our research.

## Author Contributions

YL and XQ: conceptualisation, methodology, software, validation, writing – original draft preparation, and writing – review and editing. YL: resources, formal analysis, and data curation. Both authors have read and agreed to the published version of the manuscript.

## Conflict of Interest

The authors declare that the research was conducted in the absence of any commercial or financial relationships that could be construed as a potential conflict of interest.

## Publisher’s Note

All claims expressed in this article are solely those of the authors and do not necessarily represent those of their affiliated organizations, or those of the publisher, the editors and the reviewers. Any product that may be evaluated in this article, or claim that may be made by its manufacturer, is not guaranteed or endorsed by the publisher.

## Footnotes

1 Central Administration of Customs. P. R. China. Available online: http://www.customs.gov.cn/customs/xwfb34/mtjj35/3551752/index.html (accessed on February 22, 2021).
